# Marine-Algal-Derived Postbiotics Modulating the Gut Microbiota–Adipose Tissue Axis in Obesity: A New Frontier

**DOI:** 10.3390/nu17233774

**Published:** 2025-11-30

**Authors:** Edward Kurnia Setiawan Limijadi, Kevin Christian Tjandra, Happy Kurnia Permatasari, Piko Satria Augusta, Reggie Surya, Dante Saksono Harbuwono, Fahrul Nurkolis

**Affiliations:** 1 Department of Clinical Pathology, Faculty of Medicine, Universitas Diponegoro, Semarang 50275, Central Java, Indonesia; 2 Medical Laboratory Medico Labora Purwokerto, Purwokerto 53116, Central Java, Indonesia; 3 Department of Medicine, Faculty of Medicine, Universitas Diponegoro, Semarang 50275, Central Java, Indonesia; 4 Department of Biochemistry and Biomolecular, Faculty of Medicine, Brawijaya University, Malang 65145, East Java, Indonesia; 5 School of Medicine, Faculty of Medicine, Brawijaya University, Malang 65145, East Java, Indonesia; 6 Department of Food Technology, Faculty of Engineering, Bina Nusantara University, Jakarta 11480, Jakarta, Indonesia; 7 Division of Endocrinology, Metabolism, and Diabetes, Department of Internal Medicine, Faculty of Medicine, Universitas Indonesia, Dr. Cipto Mangunkusumo National Referral Hospital, Jakarta 10430, Jakarta, Indonesia; 8 Institute for Research and Community Service, State Islamic University of Sunan Kalijaga (UIN Sunan Kalijaga), Yogyakarta 55281, Yogyakarta, Indonesia; fahrul.nurkolis.mail@gmail.com; 9 Master of Basic Medical Science, Faculty of Medicine, Universitas Airlangga, Surabaya 60131, East Java, Indonesia; 10 Medical Research Center of Indonesia, Surabaya 60286, East Java, Indonesia

**Keywords:** marine algae, postbiotics, gut microbiota, obesity, short-chain fatty acids, fucoidan, alginate, fucoxanthin, adipose inflammation, functional foods

## Abstract

**Background:** Obesity is increasingly recognized as a metabolic disorder driven by gut microbiota dysbiosis and chronic low-grade inflammation within adipose tissue. Emerging evidence highlights the gut–adipose tissue axis as a critical mediator of energy balance and metabolic regulation. Marine algae—rich in polysaccharides, polyphenols, and carotenoids—offer bioactive compounds that modulate gut microbial composition and generate beneficial metabolites termed “postbiotics.” **Objective:** This review aims to comprehensively summarize current advances in understanding how marine-algal-derived postbiotics influence the gut microbiota–adipose tissue axis and contribute to obesity prevention and management. **Methods:** A structured literature search was conducted across PubMed, Scopus, Web of Science, ScienceDirect, and SpringerLink for studies published between 2015 and October 2025. Eligible studies included in vitro, in vivo, and human trials examining the effects of marine-algal compounds on gut microbiota composition, short-chain fatty acid (SCFA) production, adipose inflammation, and metabolic outcomes. **Results:** Marine-algal polysaccharides (fucoidan, alginate, laminarin, carrageenan, and ulvan) act as fermentable fibers that enhance SCFA production and enrich beneficial taxa such as *Akkermansia*, *Lactobacillus*, and *Bacteroides*, while reducing endotoxin-producing bacteria. Polyphenols and carotenoids (fucoxanthin, phlorotannins, astaxanthin) directly target adipogenesis, oxidative stress, and adipose browning. Animal studies consistently demonstrate reduced body weight, improved insulin sensitivity, and decreased inflammation following algae supplementation. Human trials—though limited—confirm safety and show microbiota modulation with modest weight loss. **Conclusions:** Marine-algal-derived postbiotics represent a promising, natural, and sustainable strategy to target the gut microbiota–adipose tissue axis in obesity. They offer multi-targeted mechanisms through microbial and host pathways, supporting their integration into functional food and nutraceutical development. Further clinical research and regulatory standardization are warranted to translate these findings into evidence-based interventions.

## 1. Introduction

Obesity is a multifactorial metabolic disorder characterized by excess adipose tissue and chronic low-grade inflammation [1]. Global obesity data from the WHO (2022) reveals that as of 2022, 890 million adults (16% of adults worldwide, or one in eight people globally) were living with obesity, representing more than a doubling since 1990, while adolescent obesity has quadrupled over the same period. Additionally, 390 million children and adolescents aged 5–19 were overweight in 2022, including 160 million with obesity, demonstrating alarming upward trends across all age groups [2]. In recent years, research has revealed a pivotal role for gut microbiota in obesity. The trillions of microorganisms residing in our intestines influence energy balance, fat storage, and inflammation [3]. A dynamic crosstalk exists between the gut and adipose tissue, termed the gut–adipose tissue axis. Microbial metabolites and immune signals from the gut influence adipose tissue function, and vice versa [3]. In obesity, an imbalance in gut microbial composition (dysbiosis) is frequently observed, with reduced microbial diversity and a higher ratio of Firmicutes to Bacteroidetes phyla in some studies [4]. This dysbiotic state can promote greater energy harvest from diet and increased intestinal permeability, leading to elevated circulating endotoxin (lipopolysaccharide, LPS) levels that trigger inflammation in adipose tissue [5]. The outcome is adipose tissue dysfunction—marked by hypertrophic adipocytes and pro-inflammatory immune cell infiltration—which exacerbates insulin resistance and metabolic disease. Targeting this gut–microbiota–adipose axis has emerged as a promising strategy to improve metabolic health [3].

Marine algae have drawn considerable interest as potential functional foods or nutraceuticals in combating obesity and metabolic syndrome [4]. Seaweeds (marine macroalgae) have long been consumed traditionally in coastal regions, especially in East Asia, and are rich in unique fibers and bioactive compounds [4,6]. Likewise, certain microalgae (like *Spirulina* and *Chlorella*) are popular supplements known for their high nutrient and antioxidant content. Marine algae contain complex polysaccharides such as alginate, fucoidan, carrageenan, and ulvan. These compounds resist digestion and act as dietary fiber [7]. They reach the colon, where gut microbes ferment them. Algae also provide polyphenols (such as phlorotannins in brown seaweeds and bromophenols in red seaweeds), carotenoids (e.g., fucoxanthin from brown algae, astaxanthin from microalgae), and other metabolites that can directly or indirectly modulate metabolism [4,8]. Many of these compounds have demonstrated anti-obesity, anti-inflammatory, and prebiotic effects in experimental studies [8].

An intriguing concept gaining traction is that of postbiotics—beneficial bioactive compounds or components resulting from microbial fermentation or released by inactivated microbes [9,10]. Unlike live probiotic bacteria, postbiotics are non-living preparations (which may include microbial cells, cell fragments, or metabolites) that confer health benefits [9,10]. In the context of marine algae, we can consider that many of the health effects arise after algae-derived substrates interact with gut microbiota, producing metabolites (short-chain fatty acids, etc.) or modulating microbial components that improve host health [11]. Additionally, dried microalgae themselves (often consumed as inactivated powders) have been proposed to fit the definition of postbiotics since they are non-viable biomass rich in bioactives capable of health modulation [12]. Therefore, “marine-algal-derived postbiotics” in this review refer broadly to the beneficial products and effects mediated by marine-algal compounds via the gut microbiome, whether through fermentation metabolites or through algae acting as inanimate functional biomasses.

This review provides a comprehensive overview of how marine-algal-derived postbiotics modulate the gut microbiota–adipose tissue axis in the context of obesity. We first outline the literature search strategy used to gather relevant studies from the past ten years. Next, we describe the gut microbiota–adipose tissue axis and how microbial metabolites influence adipose tissue metabolism and inflammation. We then delve into the diverse types of marine algae (brown, red, green macroalgae, and microalgae) and their key bioactive components, summarizing evidence from animal models and human trials on their anti-obesity effects via gut microbiota modulation. Finally, we discuss the safety and regulatory considerations for translating marine-algal postbiotics into obesity interventions. By synthesizing recent advances, we aim to highlight marine algae as a new frontier in the fight against obesity through gut microbiome modulation.

## 2. Search Strategy

To ensure a comprehensive and up-to-date review, a structured literature search was conducted targeting studies published in the last ten years (from 2015 through October 2025). The following databases were searched, as they cover a broad range of biomedical and biological science journals: PubMed, Scopus, Web of Science, ScienceDirect, and SpringerLink. The search combined keywords related to marine algae and postbiotic effects on obesity and gut microbiota. Key terms included “marine algae,” “seaweed,” “microalgae,” “postbiotic,” “prebiotic,” “probiotic,” “gut microbiota,” “obesity,” “metabolic syndrome,” “short-chain fatty acids,” “inflammation,” and specific algal compounds or species names (e.g., “fucoxanthin,” “alginate,” “Spirulina,” “*Akkermansia*,” etc.). Search terms were combined using Boolean operators (AND, OR) and truncation. Example search string: alga* AND (obesity OR high-fat diet) AND (gut microbiota OR microbiome).

The initial database searches yielded 10,297 records. We screened titles and abstracts to identify relevant publications focusing on (1) in vitro fermentation studies of algal fibers by gut microbiota, (2) animal studies (especially rodent high-fat diet models) examining metabolic and gut microbial outcomes of algal supplementation, (3) human clinical trials or observational studies on algae intake and obesity or gut microbiome, and (4) reviews or meta-analyses on related topics. Recent review articles were used to identify additional primary studies via their reference lists. We included studies dealing with all types of marine algae (brown, red, green macroalgae; blue-green algae/microalgae) and their extracts or isolated compounds, provided they reported effects on gut microbiota composition, microbial metabolites, adipose tissue outcomes, or weight/metabolic parameters. Priority was given to studies published in peer-reviewed journals from 2013 onward to capture the latest evidence, and particularly those in the last ~5 years, reflecting the burgeoning interest in postbiotics. Non-English articles were excluded.

Data from the selected sources were extracted regarding the algae species/compound used, study design and model, key findings on gut microbiota changes (e.g., specific taxa abundance shifts, SCFA levels), and obesity-related outcomes (body weight, adiposity, inflammatory markers, etc.).

## 3. Gut Microbiota–Adipose Tissue Axis in Obesity

The gut microbiota–adipose tissue axis refers to the bidirectional communication network between intestinal microbes and adipose (fat) tissues that influences metabolic health [3]. The trillions of microbes in the gut produce a multitude of signaling molecules (metabolites, cell wall components) that can enter circulation or interact with gut neural and immune pathways, thereby affecting distant organs, including adipose depots [13]. Likewise, adipose tissue secretes hormones and inflammatory cytokines that can influence gut physiology and the microbial ecosystem. This integrative axis plays a crucial role in energy homeostasis, lipid metabolism, and systemic inflammation [3].

In obesity, perturbations in gut microbiota composition are frequently observed. Although results vary, obese individuals (and animals on high-fat diets) often exhibit a higher Firmicutes/Bacteroidetes ratio compared to lean counterparts [4]. There is also an enrichment of microbial species that harvest energy efficiently from the diet and produce compounds promoting fat storage. For example, certain Firmicutes possess enzymes that break down complex polysaccharides into absorbable sugars, potentially contributing to increased caloric extraction [4]. Moreover, dysbiosis in obesity is associated with greater production of endotoxins like lipopolysaccharide (LPS) from Gram-negative bacteria. LPS can translocate into the bloodstream (a phenomenon termed metabolic endotoxemia) when gut barrier integrity is compromised, and it binds Toll-like receptors on immune cells in adipose tissue, triggering inflammation [14]. This chronic inflammation in adipose tissue impairs insulin signaling and promotes further weight gain.

Key microbial metabolites serve as mediators in the gut–adipose axis. Short-chain fatty acids (SCFAs)—notably acetate, propionate, and butyrate—are produced by fermentation of dietary fibers by gut bacteria. SCFAs bind to receptors (like GPR43/FFAR2 and GPR41/FFAR3) on intestinal and immune cells, improving gut barrier function and modulating appetite and energy expenditure [15]. In adipose tissue, SCFAs can promote adiponectin secretion (an anti-inflammatory, insulin-sensitizing adipokine) and even stimulate browning of white adipose tissue, thereby increasing energy burning [3]. Indeed, higher circulating butyrate and propionate levels have been linked to improved metabolic profiles. Another set of mediators is bile acids, whose composition is modified by gut microbes. Secondary bile acids (like lithocholic acid and deoxycholic acid) activate receptors such as TGR5 and FXR; TGR5 activation in brown adipose tissue can enhance energy expenditure and improve glucose homeostasis. Meanwhile, microbial metabolites of dietary tryptophan (e.g., indole derivatives) interact with host receptors (like aryl hydrocarbon receptor) to reduce inflammation [16].

When the gut microbiota is in a eubiotic (healthy) state—often fostered by high-fiber diets—it produces abundant SCFAs, maintains gut barrier integrity (through butyrate’s trophic effects on colon cells and induction of mucus production), and limits LPS leakage [7]. This leads to lower systemic inflammation and healthier adipose tissue (smaller adipocytes and less macrophage infiltration). Conversely, in dysbiosis, reduced fiber fermentation and SCFA production, along with overgrowth of LPS-producing pathobionts, result in a leaky gut and pro-inflammatory milieu. The net effect is adipose tissue hypertrophy, M1-type macrophage activation in fat, and elevated adipose-derived cytokines (TNF-α and IL-6) that perpetuate insulin resistance [17].

**Figure 1:**
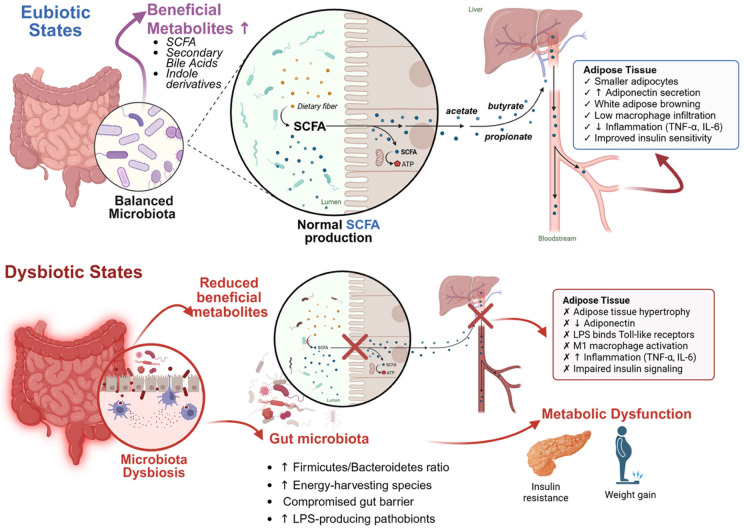
The gut microbiota–adipose tissue axis under eubiotic and dysbiotic conditions. Eubiosis: A balanced gut microbiota promotes the production of beneficial metabolites such as short-chain fatty acids (SCFAs), secondary bile acids, and indole derivatives, supporting adipose tissue homeostasis and insulin sensitivity. Dysbiosis: Imbalanced microbiota reduces beneficial metabolites, compromises gut barrier integrity (indicated by broken barrier), increases lipopolysaccharide (LPS) translocation (red arrows), and induces adipose inflammation, leading to metabolic dysfunction. Created by the authors using BioRender.com (accessed on 20 October 2025).

Critically, modulating the gut microbiota can reverse some of these pathogenic processes (Table 1 and Figure 1). Studies have demonstrated that transferring gut microbes from a lean donor or administering prebiotics/probiotics to obese models can reduce adiposity and inflammation. This has set the stage for exploring postbiotics—the beneficial factors derived from microbes—as therapeutic tools. Indeed, targeting the gut–adipose tissue axis via dietary interventions has yielded encouraging results [3]. The gut microbiome is now recognized not only as a marker but also a driver of metabolic health, making it an attractive intervention point in obesity management. Within this context, marine-algal-derived compounds are of great interest because they can beneficially shift the gut microbiome and increase production of helpful microbial metabolites. In the following sections, we examine how different types of marine algae contribute to postbiotic effects that ameliorate obesity and adipose tissue dysfunction. Conversely, a fiber-rich diet supports a diverse microbiota that produces SCFAs, strengthens the gut barrier, and reduces inflammation, thereby protecting against adipose tissue dysfunction and metabolic syndrome [3].

## 4. Marine-Algal Postbiotics: Concepts and Mechanisms

**Figure 2:**
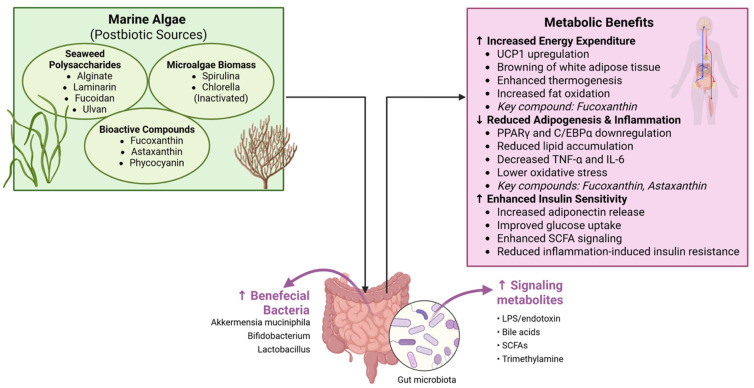
Marine algae as postbiotic sources and their metabolic benefits. Seaweed polysaccharides (alginate, laminarin, fucoidan, ulvan) and microalgae biomasses (Spirulina, Chlorella) act as postbiotic substrates that enrich beneficial bacteria (*Akkermansia*, *Lactobacillus*, *Bifidobacterium*) and enhance SCFA signaling. Bioactive compounds such as fucoxanthin, astaxanthin, and phycocyanin promote energy expenditure, reduce adipogenesis and inflammation, and improve insulin sensitivity. Created by the authors using BioRender.com (accessed on 20 October 2025).

Postbiotics are broadly defined as preparations of inactivated microbes or their components that confer health benefits to the host (Figure 2) [9]. Notably, this definition can include the complex mixture of metabolites, cell wall fragments, and compounds present after microbial fermentation or after the intentional killing of probiotic organisms [9]. Unlike probiotics (live bacteria), postbiotics do not risk translocation or infection and are not subject to viability-related safety concerns [10]. Classic examples of postbiotic factors are SCFAs, bacteriocins, peptidoglycans, and other fermentation products known to exert beneficial effects [10]. The emerging interest in postbiotics stems from their stability (i.e., they can be heated or stored without losing efficacy) and a potentially clearer regulatory path since they fall under food components rather than live cultures.

In the context of marine algae, there are two key angles to consider: 1. Algal Compounds as Prebiotics Generating Postbiotics: Many algae-derived polysaccharides are not digested by human enzymes and thus reach the colon intact. There, they serve as prebiotic substrates for gut microbes, meaning they selectively stimulate the growth or activity of beneficial bacteria. In fermenting these fibers, gut microbes produce SCFAs and other metabolites—postbiotic molecules—that improve host metabolism [36]. For instance, fermentation of alginate, laminarin, or ulvan (fibers from brown and green seaweeds) yields acetate, propionate, and butyrate, which can reduce appetite, enhance gut hormone release, and increase fat oxidation in adipose tissue [4,7]. In this way, algae act indirectly via the microbiota: the postbiotic effect is the increased SCFA and other beneficial metabolites resulting from microbial fermentation of algal prebiotics.

### Microalgae as Postbiotic Preparations

Certain microalgae (such as *Spirulina* (technically a cyanobacterium) and *Chlorella*) are often consumed as whole-biomass supplements, but in dried/inactive form. According to the ISAPP consensus, a “preparation of inanimate microorganisms” that confers health benefits qualifies as a postbiotic [9]. Although the ISAPP’s definition primarily envisioned inactivated probiotics, Martelli et al. (2025) have argued that microalgae powders could be considered postbiotic under this framework [12]. These microalgae contain bioactive nutrients (proteins, essential fatty acids, and pigments like phycocyanin) that can modulate gut microbiota and systemic health even though the algal cells themselves are not alive in the product. Indeed, *Spirulina* and *Chlorella* supplements have been shown to improve antioxidant status, reduce inflammation, and benefit lipid profiles in humans—effects comparable to those sought with postbiotics [37]. Microalgae can also be fermented by gut microbes when consumed, further releasing metabolites. Thus, microalgae blur the line between probiotic, prebiotic, and postbiotic: they are microbial (though not bacterial) biomass with innate functional properties and also substrates for the gut microbiome. Unlocking their full potential as postbiotics offers new opportunities for health and nutrition [12]. Mechanistically, marine-algal postbiotics target several aspects of the gut–adipose axis; they modify gut microbiota composition—increasing the abundance of beneficial bacteria known to correlate with leanness and metabolic health, such as *Akkermansia muciniphila*, *Bifidobacterium*, *Lactobacillus*, and certain *Bacteroides* spp., while reducing opportunistic pathogens or pro-inflammatory bacteria [4]. For example, feeding alginate oligosaccharides to obese mice boosted *Akkermansia* and *Lactobacillus*, which was linked to improved blood glucose and cholesterol levels [4]. Many algae polysaccharides are sulphated or have unique glycosidic linkages, which can enrich specific bacteria capable of degrading them, thereby reshaping the microbiota.

They enhance the production of SCFAs and other beneficial metabolites. As noted, SCFAs exert multiple metabolic benefits: acetate and propionate can signal to the brain to reduce appetite and increase fat oxidation, while butyrate improves gut barrier integrity and insulin sensitivity. Algal fibers such as fucoidan and laminarin have been shown to significantly increase fecal SCFA concentrations in high-fat-diet animals [4]. Increased SCFAs can directly affect adipose tissue by promoting the browning of white adipose (through acetate’s activation of GPR43 on adipocytes) and by increasing adiponectin release, which improves systemic insulin sensitivity.

They reduce gut permeability and inflammation. Some seaweed components stimulate mucin production or tighten junctions between gut epithelial cells. Fucoidan, for instance, alleviated gut barrier damage in obese mice, preserving the mucus layer and colon structure [4]. This prevents LPS leakage and lowers systemic inflammation. Additionally, the shift in the microbiota profile (e.g., more *Lactobacillus* and *Bifidobacterium*) tends to correlate with enhanced gut barrier function and fewer pro-inflammatory bacterial metabolites. Less LPS means adipose tissues are not being constantly triggered by endotoxin, thereby reducing inflammatory cytokine production in fat [38].

They directly deliver bioactive compounds that act on adipose tissue. Some algal-derived molecules are absorbed and travel to adipose depots. For example, the carotenoid fucoxanthin (from brown algae) or its metabolite fucoxanthinol can accumulate in fatty tissue. Fucoxanthin has been shown to upregulate Uncoupling Protein 1 (UCP1) in white adipose tissue, effectively inducing a browning effect that increases thermogenesis (heat generation) and fat burning [8]. It also downregulates adipogenic genes (PPARγ and C/EBPα) and reduces lipid accumulation in developing fat cells [8]. These direct actions complement the microbiota-mediated effects, leading to reduced adiposity. Similarly, astaxanthin from microalgae is a potent antioxidant that can dampen adipose inflammation. Astaxanthin supplementation in high-fat diet models lowers oxidative stress in adipose tissue and shifts the gut microbiota toward a healthier profile (restoring the Firmicutes/Bacteroidetes balance, increasing carbohydrate-utilizing beneficial microbes) [8]. The antioxidant and anti-inflammatory actions of astaxanthin in fat depots contribute to improved insulin sensitivity and less weight gain, illustrating a combined direct + microbiota-mediated mechanism. Overall, marine-algal postbiotics operate through multi-faceted mechanisms: they feed good bugs, starve or suppress bad bugs, fortify the gut barrier, reduce inflammatory signals, and supply bioactives that target adipose metabolism. By doing so, they help realign the gut–adipose axis toward a healthier state (higher SCFAs and adiponectin, lower LPS and TNF-α, more energy expenditure).

## 5. Marine-Algal Bioactive Compounds and Their Gut Microbiota-Mediated Anti-Obesity Effects

### 5.1. Brown Seaweeds (Phaeophyceae)

**Figure 3:**
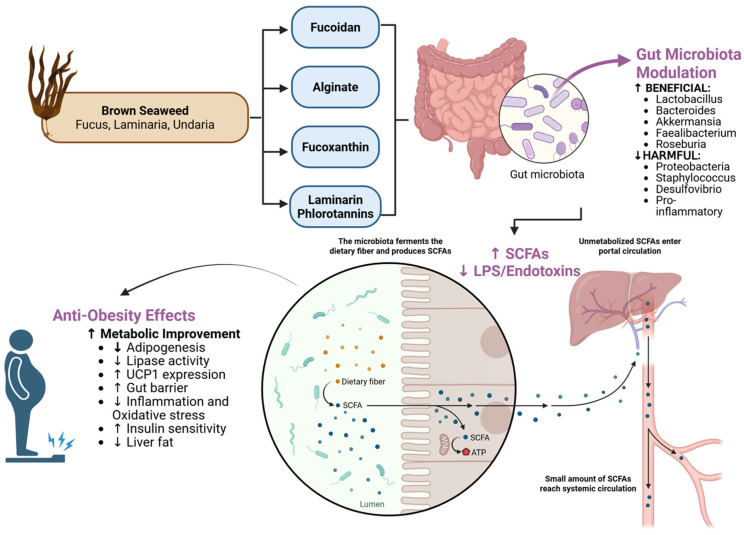
Brown seaweed-derived compounds and their microbiota-mediated anti-obesity mechanisms. Top: Major bioactive compounds from brown algae (fucoidan, alginate, laminarin, phlorotannins). Middle: Effects on gut microbiota showing increased beneficial taxa (*Lactobacillus*, *Bacteroides*, *Akkermansia*) and decreased harmful taxa (*Proteobacteria*, *Staphylococcus*). Bottom: Downstream metabolic effects, including increased SCFA production (acetate, propionate, butyrate) and decreased LPS/endotoxin levels. The resulting improvements in gut barrier function, reduced inflammation, and enhanced adipose metabolism contribute to overall metabolic health. Created by the authors using BioRender.com (accessed on 20 October 2025).

Brown algae are perhaps the most intensively studied for anti-obesity effects, owing to their abundance of unique polysaccharides and pigments (Figure 3). Fucoidan, alginate, laminarin, and fucoxanthin are hallmark compounds from brown seaweeds like *Fucus vesiculosus*, *Ascophyllum nodosum*, *Laminaria*/*Saccharina* (kelps), and *Undaria pinnatifida* (wakame) [39]. The commercial recovery of these bioactives varies considerably in cost and feasibility: alginate (USD 5–20/kg) is the most economical due to well-established industrial extraction processes, while fucoidan (USD 50–150/kg), phlorotannins (USD 100–400/kg), and fucoxanthin (USD 200–800/kg) require more complex extraction procedures that increase costs [40,41,42]. Although these recovery costs currently exceed those of terrestrial plant extracts, integrated biorefinery approaches that simultaneously co-extract multiple compounds, combined with emerging technologies such as enzyme-assisted and supercritical fluid extraction, are projected to reduce costs by 30–50% within the next decade, thereby improving commercial viability for both functional food and nutraceutical applications [43].

**Fucoidan**: This is a sulfated fucose-rich polysaccharide found in brown algal cell walls. Multiple animal studies have shown that fucoidan supplementation attenuates weight gain and adipose tissue expansion in high-fat diet (HFD) mice [4]. Fucoidan improves blood lipid profiles and reduces liver fat accumulation [44]. Part of its anti-obesity mechanism is through gut microbiota modulation: fucoidan supplementation consistently reverses the elevated Firmicutes/Bacteroidetes ratio observed in HFD-induced obesity, bringing it closer to a lean-like profile [4]. Fucoidan-fed obese mice had increased relative abundance of *Lactobacillus, Faecalibacterium, Blautia, Alistipes, Ruminococcus*, and *Bacteroides*—genera known to be negatively correlated with obesity [45]. Concurrently, fucoidan decreased the abundance of bacteria associated with obesity and inflammation, such as *Staphylococcus, Streptococcus, Oscillospira, Lachnoclostridium*, and *Desulfovibrio*. Treatment enriched *Akkermansia muciniphila* (see Table 1), contributing to improved gut barrier integrity [46]. The expansion of *Akkermansia* is thought to improve gut barrier integrity and reduce endotoxemia. Fucoidan significantly increased fecal SCFA concentrations, particularly acetate, propionate, and butyrate [47]. These SCFAs contribute to appetite regulation and improved insulin sensitivity, thereby aiding weight control. Furthermore, fucoidan alleviated HFD-induced gut mucosal damage (preventing colon shortening and mucosal disruption)—likely a result of enriched beneficial microbes that promote intestinal integrity [48]. Through these concerted actions, fucoidan supplementation in animals leads to smaller adipocytes and less adipose inflammation. Although human studies on fucoidan for obesity are still scarce, the robust preclinical data have spurred interest in its use as a functional ingredient. Indeed, fucoidan is non-toxic and in some countries is sold as a dietary supplement. Its dual role as fiber (prebiotic) and bioactive (antiangiogenic, anti-inflammatory) makes it a compelling candidate in obesity management [49].

**Alginate**: Brown seaweeds are rich in alginates—viscous polysaccharides composed of mannuronic and guluronic acid units. Sodium alginate is widely used as a food additive and fiber supplement. In the stomach, alginate forms a gel, promoting satiety and reducing the post-meal glycemic response [8]. But beyond its physical effects, alginate has notable prebiotic properties in the colon. Alginate oligosaccharides (obtained by breaking down alginate) fed to obese mice significantly improved metabolic profiles and insulin sensitivity, accompanied by a remodeling of gut microbiota [50]. Specifically, unsaturated alginate oligosaccharides (UAOSs) partly reversed HFD-induced dysbiosis: they increased the phylum Bacteroidetes and Actinobacteria while decreasing Proteobacteria (often overgrown in obesity) [4,50]. At a finer scale, UAOSs selectively enriched *Lactobacillus* and *Akkermansia* and reduced pro-inflammatory genera like *Proteus* and *Enterobacteriaceae* [4]. Another study on low-molecular-weight alginate found increased *Bacteroides* (beneficial fiber-degraders) and decreased *Lachnospiraceae* (some of which are LPS producers) in treated obese mice [51]. Both studies noted that alginate treatment led to higher cecal SCFA concentrations, indicating enhanced fermentative activity of the microbiota [51]. These microbial changes translated into tangible metabolic benefits: alginate-fed mice had lower weight gain, reduced body fat accumulation, and improved markers of inflammation and lipid metabolism [50,52]. In humans, alginate has shown promise as well. A double-blind randomized trial in overweight adults (15 g/day alginate for 14 weeks) demonstrated modest but significant weight loss compared to placebo [52]. The weight reduction was attributed not only to increased satiety but also to changes in bile acid metabolism and gut microbiota observed in participants. Another human study found that alginate supplementation led to increased fecal bile acid excretion and altered gut microbiome composition, which was correlated with the degree of weight loss [53]. Researchers believe alginate’s gel-forming fiber drives shifts toward bacteria that produce succinate and propionate, metabolites that can stimulate intestinal gluconeogenesis and satiety signals. It is also believed that alginate helps prevent weight gain by improving the gut microbiota—an assertion supported by recent findings [50,51]. Importantly, alginate’s long track record in foods (e.g., as a thickener) means it is generally recognized as safe. Thus, alginate represents a readily translatable algae-derived prebiotic that yields postbiotic benefits (SCFAs and beneficial taxa) to counter obesity [54]. Other brown algal fibers, such as laminarin, similarly increased beneficial SCFA-producing bacteria and reduced pathogenic bacteria, leading to reduced weight gain and inflammation. These microbial changes lead to increased SCFA production and lower LPS, thereby improving adipose tissue function and insulin sensitivity [55].

**Laminarin**: A smaller glucan fiber from brown algae, laminarin has also shown prebiotic effects. In a study by Nguyen et al. (2016), laminarin supplementation in HFD-fed mice significantly decreased weight gain and adiposity [56]. This was accompanied by an improved Firmicutes/Bacteroidetes ratio and increased abundance of *Clostridium cluster XIVa, Bacteroides*, and *Parabacteroides*—groups of bacteria often linked to healthy fermentation and leanness. Pathogenic Clostridium clusters (XIVb, XI) were reduced [56]. Interestingly, laminarin also increased gut microbial genes encoding carbohydrate-active enzymes, indicating enhanced capability to break down complex carbs [4,56]. The result is likely more SCFA production and energy excretion (some studies noted increased fecal energy losses with laminarin, suggesting less calorie absorption). Although laminarin is less abundant than alginate in seaweeds, it could be a potent additive when extracted.

**Fucoxanthin**: This is an orange-brown carotenoid that gives brown seaweeds their color. Fucoxanthin has garnered attention for its unique anti-obesity actions. It can stimulate UCP1 expression in white adipose tissue, effectively converting some white fat into a more metabolically active, thermogenic state (similar to brown fat) [8]. This increases energy expenditure and reduces fat accumulation. Fucoxanthin also suppresses adipocyte differentiation and lipid accumulation in cells by downregulating adipogenic transcription factors (like PPARγ) [57]. In mice, fucoxanthin supplementation (typically via fucoxanthin-rich wakame seaweed or extracts) has repeatedly shown a reduction in body weight gain and visceral fat mass despite animals being on high-fat diets [8,58]. Part of fucoxanthin’s effect is systemic (via gene regulation), but it also influences the gut microbiome. For instance, a 2020 study found that fucoxanthin-fed obese mice experienced shifts in gut microbiota, including increases in *Lactobacillaceae* and *Roseburia* (butyrate-producing bacteria) and decreases in pro-inflammatory taxa. These changes were associated with reduced plasma LPS and inflammatory cytokines [59]. Another study noted fucoxanthin raised levels of *Akkermansia* and *Bacteroidetes* while lowering *Firmicutes* and *Proteobacteria*, thus improving the F/B ratio [60]. The evidence suggests that fucoxanthin may act in part by favoring a microbiome that is characteristic of lean phenotypes. Human data on fucoxanthin is emerging. A small clinical trial in 2023 (28 patients with metabolic syndrome) tested 12 mg/day fucoxanthin vs. placebo for 12 weeks [61]. The fucoxanthin group saw significant reductions in body weight (~1.5 kg), BMI, and waist circumference, as well as lowered blood pressure and triglycerides [61]. Notably, insulin sensitivity improved, and insulin secretion metrics were better, indicating metabolic benefits beyond weight loss [61]. While that study did not measure gut microbes, it adds clinical credence to fucoxanthin’s anti-obesity potential. Fucoxanthin is available in some dietary supplements (often combined with oils for absorption). Regulatory approval as a novel food ingredient is underway in certain regions. Altogether, fucoxanthin stands out as an algae-derived molecule that can independently act on adipose tissue and also modulate the gut–adipose axis via microbiome changes.

**Phlorotannins**: These polyphenolic compounds from brown seaweeds (e.g., *Ecklonia* species) deserve mention. Phlorotannins, such as dieckol and fucophlorethol, have anti-diabetic and anti-obesity effects by inhibiting digestive enzymes like pancreatic lipase [62]. They may reduce dietary fat absorption and thus limit weight gain. Phlorotannins also have prebiotic-like actions—being biotransformed by gut bacteria into smaller phenolics that can exert local antioxidant and anti-inflammatory effects in the gut. Some studies have shown phlorotannin extracts increase *Bifidobacterium* counts in vitro, though in vivo evidence is still limited (the Frontiers review noted that phlorotannins have anti-metabolic syndrome functions, but few studies have detailed their microbiota effects) [4]. Nonetheless, given their enzyme inhibition and antioxidant capacity, phlorotannins likely contribute synergistically to the anti-obesity effect of whole seaweed.

In summary, brown seaweeds provide a multitude of compounds that tackle obesity on several fronts. Through fibers like fucoidan, alginate, and laminarin, they beneficially reshape the gut microbiota (more SCFAs, higher *Akkermansia/Lactobacillus*, and lower endotoxins), while through bioactives like fucoxanthin and phlorotannins, they directly influence adipose tissue metabolism and nutrient absorption. Animal and early human trials strongly support the potential of brown algae in obesity intervention, establishing a foundation for translational research and development of functional foods or nutraceuticals.

### 5.2. Red Seaweeds (Rhodophyta)

**Figure 4:**
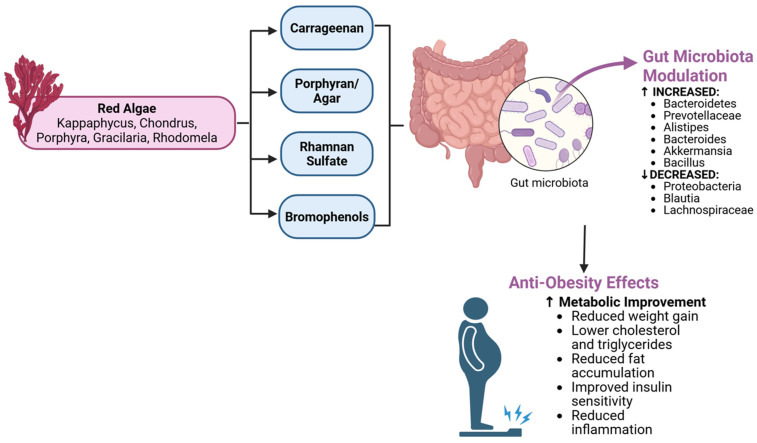
Gut microbiota modulation and anti-obesity effects of red algae. Key polysaccharides (carrageenan, porphyran, rhamnan sulfate) and bromophenols from *Kappaphycus*, *Gracilaria*, *Porphyra*, and *Rhodomela* modulate gut microbiota composition by increasing beneficial taxa (*Bacteroidetes*, *Akkermansia*) and decreasing harmful ones (*Proteobacteria*, *Blautia*), leading to reduced weight gain, improved insulin sensitivity, and decreased inflammation. Created by the authors using BioRender.com (accessed on 20 October 2025).

Red algae are another diverse group with distinctive polysaccharides such as carrageenans, agar/agarose, and porphyran, as well as unique polyphenols (e.g., bromophenols) (Figure 4). While red seaweeds are less extensively studied for obesity than brown algae, emerging research indicates they, too, can modulate the gut microbiome in ways that combat metabolic dysfunction.

**Carrageenan**: This is a sulfated galactan commonly extracted from red seaweeds like *Kappaphycus alvarezii* and *Chondrus crispus*. Carrageenan is widely used as a thickener in foods. It comes in several forms (κ, ι, and λ), with κ-carrageenan being prevalent. Notably, carrageenan’s reputation in research is mixed—high doses of *degraded* carrageenan can induce inflammation in the gut (used in some models of colitis). However, food-grade (undegraded) carrageenan and certain doses may have metabolic benefits. Two recent mouse studies found that κ-carrageenan supplementation in HFD-fed mice prevented weight gain, lowered cholesterol and triglycerides, and reduced fat accumulation [63]. These improvements coincided with significant shifts in the gut microbiota: carrageenan-treated obese mice showed a normalization of the phylum ratio imbalances—specifically, a decrease in the obesity-associated phylum Proteobacteria and an increase in Bacteroidetes [4]. At the family/genus level, beneficial groups like *Prevotellaceae*, *Alistipes*, and *Bacteroides* were enriched, and these correlated negatively with blood lipid levels [4,64]. Meanwhile, carrageenan reduced the abundance of *Blautia, Lachnospiraceae*, and *Erysipelotrichaceae*—bacterial groups positively correlated with weight gain and hyperglycemia [4]. This suggests carrageenan’s fiber effect can selectively promote bacteria that improve host metabolism (e.g., *Alistipes* is linked to leanness, *Prevotella* to fiber fermentation). There is a bit of a paradox since some earlier studies have suggested carrageenan could impair glucose tolerance, but those often involve high concentrations and specific models. The newer studies point toward a dose window where carrageenan acts as a prebiotic. In one mouse study, carrageenan even improved insulin sensitivity and reduced inflammatory markers, aligning with the observed microbial changes [65]. It is clear that more research is needed to reconcile these effects and determine safe, efficacious ranges. Human data on carrageenan and microbiota are limited; however, a clinical trial is currently investigating whether dietary carrageenan impacts the human gut microbiome [66]. Given carrageenan’s prevalence in the food supply, understanding its role as a potential metabolic modulator is important.

**Porphyran and Agar**: Porphyran is a sulfated polysaccharide from *Porphyra* (nori seaweed), and agarose (component of agar) comes from various red algae (*Gelidium* and *Gracilaria*). These fibers are also largely indigestible by humans but fermentable by gut microbes. Porphyran in particular has shown antidiabetic and anti-inflammatory effects in preliminary studies. A study in *Drosophila* and mice noted that porphyran from *Pyropia yezoensis* could ameliorate metabolic abnormalities; in fruit flies, porphyran reduced sugar levels and changed the microbiota by decreasing pathogenic *Proteobacteria* (e.g., *Escherichia/Shigella*) and increasing beneficial *Bacillus* and *Akkermansia* [67,68]. This suggests that even in simple models, porphyran fosters a gut environment protective against metabolic stress. In mammals, agar-type fibers are known to increase stool bulk and potentially bind some lipids, but their effect on microbiota is less documented. One can surmise that certain gut bacteria (like *Bacteroides* uniformis, which can degrade porphyran) thrive on these polysaccharides, which could have downstream benefits such as SCFA production and gut barrier enhancement. Further research is needed, but red algal fibers hold promise as novel prebiotics. Recent in vitro and in vivo studies have further elucidated the prebiotic- and postbiotic-generating potential of red seaweeds. Lopez-Santamarina and colleagues (2025) demonstrated that red seaweeds, including Palmaria palmata, Porphyra umbilicalis, and Chondrus crispus, exert modulatory effects on human gut microbiota composition via fermentation, leading to the production of beneficial postbiotic metabolites [69]. Their in vitro model showed that these red algae selectively promoted the growth of beneficial bacteria while simultaneously generating short-chain fatty acids and other bioactive metabolites that could influence host metabolism. Das et al. (2023) characterized the phytochemical constituents and bioactivity of *Kappaphycus alvarezii*, a commercially important red seaweed [70]. They identified diverse antimicrobial properties and bioactive compounds that not only directly influence pathogenic bacteria but also serve as substrates for probiotic bacteria, thereby facilitating postbiotic production in the gut environment. This dual action—antimicrobial activity against pathogens combined with prebiotic effects for beneficial microbes—exemplifies the sophisticated mechanisms by which red seaweeds modulate the gut–adipose axis. Similarly, Ismail et al. (2023) documented the nutritional profile and antioxidant capacity of brown seaweeds from the Red Sea, noting high levels of polyphenolic compounds and unique polysaccharides [71]. Although focused on brown species, their findings parallel those for red algae, suggesting that diverse marine macroalgae share common mechanisms of metabolic modulation through both direct bioactive delivery and indirect microbiota-mediated postbiotic generation. These studies collectively support the concept that red seaweeds function as both prebiotic substrates and sources of bioactive compounds that work synergistically to promote metabolic health through the gut microbiota–adipose tissue axis.

**Rhamnan Sulfate (RS)**: Though often associated with green algae (like *Monostroma*), some red algae also contain similar sulfated polysaccharides. Interestingly, a study by Shimada et al. used a rhamnan sulfate from seaweed on both mice and a small human trial [4,72]. In HFD-fed mice, RS increased fecal bulk and energy excretion, alleviating metabolic syndrome features. When a 2-week pilot trial in humans with infrequent bowel movements gave 100 mg/day of RS, stool frequency increased (suggesting improved laxation), and the gut microbiota shifted toward more Bacteroidetes and fewer Clostridia [4,73]. While body weight did not change in 2 weeks (expectedly), these results highlight that sulfated algal polysaccharides can modulate microbiota even in humans, potentially aiding gut health and indirectly metabolism. RS seemed to increase certain orders like Negativicutes, which are linked to constipation relief, and raised acetic acid producers that can stimulate peristalsis [72]. This indicates a prebiotic action consistent with other algae fibers. Such sulfated rhamnans could thus be valuable both for gastrointestinal regulation and metabolic outcomes over a longer term.

**Bromophenols and Other Phenolics**: Marine red algae produce unique halogenated phenolic compounds. One example is a bromophenol (BDB) isolated from *Rhodomela confervoides*, which was found to alleviate type 2 diabetes in mice while modulating gut microbiota [74]. Although not directly an obesity study, it hints that red algal phenolics can influence gut microbes (possibly by inhibiting certain bacteria or acting as antioxidants) and improve metabolic parameters. Red algal polyphenols have not been as deeply explored as those from brown algae, but given the potent bioactivity of some (e.g., anti-adipogenic effects in cell studies), they likely contribute to the overall effect of consuming red seaweeds.

Red seaweeds like *Gracilaria*, *Eucheuma*, and *Palmaria* are also rich in protein and minerals, which can have ancillary benefits. Some red algae are traditional foods (e.g., dulse and nori). Epidemiological observations in Asia suggest that regular seaweed consumption correlates with lower obesity prevalence, although confounders exist [75,76]. Animal studies collectively suggest red algal fibers can act as prebiotics to reduce hyperlipidemia and weight gain, mainly by altering the gut microbiota toward more fermentative, less endotoxin-producing communities [4,8]. Red algae merit more research, especially human trials, to fully establish their postbiotic potential in obesity.

### 5.3. Green Seaweeds (Chlorophyta)

Green macroalgae, such as *Ulva* (sea lettuce), *Codium*, and *Monostroma*, contain their own array of functional compounds, including ulvan (sulfated rhamnose-rich polysaccharide), various sulfated heteropolysaccharides, and even unique pigments like siphonaxanthin.

**Ulvan and Sulfated Polysaccharides**: Ulvan is a major polysaccharide in *Ulva* species, composed of sulfated sugars (rhamnose, xylose, glucuronic acid). Like other algae fibers, ulvan is fermentable by gut bacteria. Although specific obesity studies with ulvan are few, one can extrapolate from metabolic syndrome models: ulvan has shown cholesterol-lowering and anti-inflammatory effects in rodents, presumably via binding bile acids and altering gut flora. A 2021 study (Li et al.) reported that sulfated polysaccharides from *Codium fragile* (a green seaweed) had prebiotic and anti-obesity effects, increasing populations of *Bacteroidetes* and SCFA producers in the gut and reducing body weight gain in mice [77,78]. The treated mice had improved insulin sensitivity and less liver fat, linked to changes in gut microbial metabolites. These findings align with the idea that green seaweed fibers, like their brown and red counterparts, can modulate the microbiome beneficially.

**Rhamnan Sulfate**: As mentioned earlier, Monostroma-derived rhamnan sulfate has been studied for gut health effects. Green algae are a primary source of RS. The preliminary human data showing increased defecation frequency and microbiota shifts with RS supplementation underscores the fiber’s activity [79]. Over a longer term, such improvements in gut function could translate into metabolic benefits, considering that alleviating constipation and increasing SCFA (like acetate) can influence hormones such as PYY and GLP-1 that regulate appetite.

**Siphonaxanthin**: A rare carotenoid from green algae (notably *Codium* and *Caulerpa*), siphonaxanthin has shown anti-obesity properties in cell and animal models. It appears to inhibit adipogenesis—in 3T3-L1 adipocyte cells, siphonaxanthin prevented lipid accumulation and downregulated adipogenic genes (C/EBPα, PPARγ, and SREBP-1c) [8]. It may also stimulate genes related to energy expenditure in adipose tissue. While its effects on gut microbiota have not been reported in detail, any compound affecting systemic metabolism could indirectly influence the gut (for instance, by altering bile secretion or inflammatory tone in the intestine) [80]. Siphonaxanthin is an example of a bioactive that could work in concert with algae fibers to produce an anti-obesity effect.

**Proteins and Peptides**: Green seaweeds can be relatively high in protein (for example, *Ulva* has all essential amino acids). During fermentation by gut microbes, proteins can be broken down into peptides, some of which might act as signaling molecules or modulate microbial composition (though excessive protein fermentation is usually not beneficial, leading to putrefactive metabolites). However, specific peptides from algae (e.g., derived via enzymatic hydrolysis) have shown cholesterol-lowering or anti-inflammatory effects in vitro [81]. One study found that a protease hydrolysate of *Spirulina* (a cyanobacterium often grouped with microalgae) improved lipid metabolism and gut microbiota in obese mice, increasing *Lactobacillus* and reducing *Desulfovibrio* counts [82]. While *Spirulina* is not a green seaweed, this highlights that algae proteins can influence the microbiome milieu.

In general, green seaweeds are less studied than brown, but available evidence suggests that their sulfated polysaccharides act similarly to those of brown and red algae, fostering beneficial gut bacteria and metabolic improvements. Additionally, unique compounds like siphonaxanthin add a direct anti-obesity mechanism. Green algae often have a mild taste and have been incorporated into certain functional foods (e.g., Ulva in salads, Codium in extracts)—meaning that there is potential for their expanded use if efficacy is confirmed. For instance, a trial of *Ulva* supplementation in humans could assess weight and microbiome changes, something yet to be published to our knowledge.

### 5.4. Microalgae and Cyanobacteria

This category includes photosynthetic microorganisms such as *Spirulina* (Arthrospira), *Chlorella*, *Dunaliella*, and the alga *Haematococcus pluvialis* (source of astaxanthin). Microalgae are usually consumed as whole-biomass supplements (pills, powders, or added to foods). They are rich in protein, polyunsaturated fatty acids (PUFAs like gamma-linolenic acid in Spirulina), vitamins, minerals, and pigments (phycocyanin in Spirulina, chlorophyll, carotenoids, etc.). As discussed earlier, dried microalgae can be considered a form of postbiotic in themselves [12].

***Spirulina*** (Arthrospira): Spirulina is a blue-green filamentous cyanobacterium commonly sold as a nutraceutical. It has demonstrated significant anti-obesity effects in both preclinical and clinical settings. A 2019 systematic review and meta-analysis of randomized trials concluded that *Spirulina* supplementation leads to a significant reduction in body weight (on average, ~1.56 kg vs. controls), with even greater effects on obese individuals (about 2 kg of weight reduction) [37]. Spirulina also reduced body fat percentage and waist circumference in these analyses [37]. These clinical outcomes make Spirulina stand out among microalgae for weight management. In terms of mechanism, Spirulina is not a fiber source, but rather, its components act on metabolism and possibly the microbiome. Animal studies indicate Spirulina can modulate the gut microbiota: for example, Spirulina feeding in high-fat-diet rats shifted the microbiome composition and reduced intestinal permeability and inflammation [83]. One study showed *Spirulina* alleviated chronic inflammation by modulating gut microbiota—it increased beneficial genera and tightened gut junctions, thereby lowering systemic LPS and inflammation in HFD-fed rats [83]. Another study attributed Spirulina’s benefits to its polyunsaturated fatty acid content, which the gut microbes might convert into anti-inflammatory metabolites [84]. Moreover, Spirulina’s phycocyanin pigment has been shown to have hepatoprotective and antioxidant effects, which could indirectly influence gut–adipose signaling by reducing oxidative stress signals. Clinically, Spirulina appears to improve lipid profiles and glycemic control and also may suppress appetite (some users report reduced cravings, possibly due to its high protein content influencing satiety hormones) [85]. So, while Spirulina’s direct impact on gut microbiota in humans is not fully characterized, it likely contributes to a more anti-inflammatory gut environment. Given its solid evidence base, Spirulina can be considered a microalgal postbiotic with anti-obesity efficacy.

***Chlorella***: Another popular microalga, Chlorella is a green unicellular alga. Some RCTs have found that Chlorella supplementation yields modest weight loss and improvements in cholesterol and blood glucose. Chlorella contains a good amount of fiber in its cell wall (though broken cell formulations are used for digestibility), which can have prebiotic effects. A study on overweight adults found that 12 weeks of Chlorella intake led to reductions in body fat percentage and fasting blood sugar, and it was suggested that changes in gut hormones were involved. Chlorella might also increase SCFA-producing bacteria due to its fibers and resistant proteins, though dedicated microbiome analyses are limited [86]. Still, as a nutrient-dense supplement, Chlorella likely supports metabolic health in part via gut modulation.

**Astaxanthin from *Haematococcus***: We touched on astaxanthin in the context of red/brown algae, but commercial astaxanthin is actually produced by the microalga *Haematococcus pluvialis*. Astaxanthin is often marketed for antioxidant and anti-inflammatory benefits. In obesity, astaxanthin has been shown to reduce fatty liver and improve lipid profiles in animal models [87]. Remarkably, it also alleviated gut dysbiosis in HFD mice, restoring the Firmicutes–Bacteroidetes ratio and increasing counts of beneficial microbes associated with improved carbohydrate/lipid metabolism [79]. It reduced *Desulfovibrio* and other endotoxin producers while promoting bacteria that may contribute to SCFA production and gut homeostasis [88]. These microbiota changes, combined with astaxanthin’s reduction in oxidative stress in adipose tissue, resulted in less weight gain and improved insulin sensitivity in those studies [88]. Human data is nascent; one study on humans suggested that astaxanthin supplementation can improve HDL cholesterol and reduce oxidative stress markers, but more research on weight outcomes is needed. Nonetheless, astaxanthin stands as a potent example of a single microalgal metabolite yielding postbiotic-like effects (because it alters microbial composition indirectly by reducing inflammation and possibly by being used by certain bacteria).

**Other Microalgae**: Species like *Dunaliella salina* (rich in β-carotene) and *Nannochloropsis* (rich in EPA omega-3 fatty acid) have not been studied in an obesity context directly, but their components are known to benefit metabolism (e.g., omega-3s from algal oil reduce inflammation and could shift gut microbiota toward more anti-inflammatory profiles). It is plausible that as research progresses, more microalgae will be recognized for their prebiotic or postbiotic contributions [89].

In aggregate, microalgae offer a different angle: they are often consumed for their dense nutrition and bioactive molecules, and these in turn modulate host metabolism and possibly gut microbiota indirectly. Unlike macroalgal fibers, microalgae do not provide bulk fermentable polysaccharides to feed gut bacteria (except for whatever fiber is in their cell walls), but they can still shape the gut ecosystem via antimicrobial or growth-promoting activities. For instance, Spirulina has mild antimicrobial activity against some pathogenic bacteria, potentially allowing beneficial commensals to thrive. Microalgae can also bind endotoxins; there is some evidence that Spirulina can bind LPS and prevent it from inducing inflammation, effectively acting as an LPS scavenger. This could improve the gut environment for beneficial microbes. Thus, microalgae complement macroalgae in a comprehensive obesity intervention: macroalgae predominantly provide fermentable substrates to generate postbiotics like SCFAs, while microalgae provide functional nutrients and direct postbiotic substances that reduce oxidative stress and inflammation.

## 6. Evidence from Human Trials

Translating the promising effects of marine-algal postbiotics from bench to bedside is critical. While human studies are still relatively limited compared to animal research, a number of clinical trials and interventions have been conducted in the last decade, demonstrating the safety and potential efficacy of marine algae in modulating gut health and obesity-related outcomes. Notable human evidence includes the following:

**Whole-Seaweed Supplementation (Prebiotic Approach):** A pioneering randomized controlled trial in 2025 tested daily consumption of the brown seaweed *Himanthalia elongata* (sea spaghetti) in overweight adults [7]. Participants took 2 g of encapsulated whole seaweed per day for 30 days. Results: The seaweed group exhibited significant changes in gut microbiota composition, notably increases in SCFA-producing bacteria such as *Parabacteroides distasonis, Bacteroides eggerthii, B. uniformis*, and others, compared to placebo [7]. These shifts suggest enhanced fiber fermentation and a more metabolically favorable microbiome. The study provided the first clinical evidence that even a small amount of whole brown seaweed can beneficially modulate the human gut microbiota [7]. While 30 days was short and no major weight loss occurred (likely due to the brief duration and only 2 g dose), the improvement in microbiota and SCFA profiles supports the use of seaweed as a functional prebiotic ingredient in diets aimed at better metabolic health [7]. Indeed, the authors concluded that *H. elongata* could serve as a novel prebiotic to boost gut health in overweight individuals [7]. This study validates in humans what many animal studies have shown—that brown seaweed fiber can nurture beneficial gut microbes with downstream health effects.

**Alginate Trials:** As noted earlier, alginate (from brown algae) has been tested in human weight loss studies. In one trial, overweight participants were given alginate supplements (either as powder or incorporated into foods like bread) over several weeks. A key finding was that alginate supplementation led to greater weight loss compared to the control, and importantly, it was associated with changes in gut microbiota and increased fecal bile acid excretion [90]. The increased excretion of bile acids suggests that alginate binds bile in the gut, causing the body to use more cholesterol to synthesize new bile acids—thus lowering cholesterol and possibly altering microbial bile acid metabolism in the colon. Additionally, a 2023 double-blind trial in China reported that alginate intake improved body weight and BMI and impacted the gut microbiome composition (though full results are pending publication). These trials underscore that alginate—easily consumed as a fiber supplement—not only aids in weight control via appetite and fat absorption mechanisms, but likely via shaping the gut ecosystem (e.g., promoting *Bacteroides* that thrive on polysaccharides, etc.) [90].

**Spirulina and Microalgae in Humans:** Multiple clinical trials have shown that Spirulina leads to weight and fat loss. For example, one RCT found that obese patients who took 2 g/day Spirulina for 3 months lost significantly more weight and had a reduction in waist circumference compared to a placebo [37]. In another trial, Spirulina (along with a low-calorie diet) improved BMI and leptin levels more than diet alone. While gut microbiota was not measured in these studies, the anti-obesity outcomes align with animal data where Spirulina modulated gut bacteria and inflammation. Some small trials also reported that *Chlorella* intake for 8–12 weeks improved fat percentages and lowered fasting glucose in participants with fatty liver or metabolic syndrome, implicating improvements in metabolic endotoxemia or gut hormone signaling. A recent pilot study combined Spirulina with exercise in overweight adults and found greater reductions in body fat and CRP (inflammatory marker) than exercise alone [91], hinting at an inflammation-lowering effect, perhaps via gut modulation.

**Fucoxanthin Supplements:** A product containing brown seaweed extract (with fucoxanthin) plus pomegranate seed oil was tested in a 16-week trial in women with obesity (often cited in earlier reviews). Though that study was about a decade ago (slightly outside our 10-year window), it famously showed significant reductions in body weight and liver fat in the fucoxanthin group. More recently, the 2023 trial from Mexico we described found that isolated fucoxanthin (12 mg/day) led to mild weight loss and improved metabolic syndrome criteria [61]. There is ongoing interest in developing fucoxanthin as a nutraceutical—Japan has approved a fucoxanthin-rich oil as a “Food for specified health use” (FOSHU) for weight and fat reduction. In terms of gut microbiota, an unpublished but intriguing human study is the “XANADU” trial, which is examining fucoxanthin’s effect on gut microbiome in overweight individuals (results pending). The expectation is that fucoxanthin may increase the relative abundance of beneficial gut microbes in humans, similar to animals, though confirmation is needed.

**Seaweed-Enriched Foods:** Some trials have incorporated seaweeds into regular foods to test acceptability and effects. In one study, participants consumed bread enriched with 4% seaweed (Ascophyllum nodosum) daily for 6 weeks. The seaweed-bread group had reductions in energy intake (possibly due to increased satiety from fiber and umami flavor) and a trend toward improved gut bacteria profile (with more bifidobacteria reported). In another trial from Ireland, adding kelp to the diet improved iodine status but required careful monitoring to avoid excess (discussed under safety). These functional food approaches show promise for integrating algae into diets conveniently [92,93].

Overall, human trials, though fewer in number, consistently indicate that marine algae and their extracts are well tolerated and can positively influence weight-related outcomes and gut microbiota in people (Table 2). The weight losses observed (typically 1–2 kg over a few months) might seem moderate, but even this magnitude, if accompanied by reduced inflammation and better lipid/glucose levels, is clinically meaningful in metabolic syndrome management. Importantly, many of these interventions also report improvements in subjective well-being, digestion, and reduction in hunger/appetite—all beneficial for long-term adherence. It should be noted that individual responses vary; some people may harbor gut microbiomes more or less receptive to a given algal fiber (a concept known as “enterotype-specific” response). Future personalized nutrition approaches might tailor which type of algae is best for a person’s microbiome profile. To sum up, human evidence is mounting that marine-algal postbiotics are not just a lab curiosity but a tangible tool. As research continues, we anticipate larger and longer trials will cement their role, perhaps even leading to approved health claims (e.g., “seaweed fiber X reduces risk of type 2 diabetes by improving gut health”). The challenge will be to ensure consistency of products (algal composition can vary by harvest) and palatability (overcome seaweed’s strong taste in some cases). Encouragingly, products like kelp noodles, algae snacks, and microalgae smoothies are increasingly available, reflecting a trend toward embracing these “ocean superfoods” for metabolic wellness.

## 7. Regulatory and Safety Considerations

As marine-algal postbiotics advance from research to real-world application, it is crucial to address regulatory status and safety. Marine algae have been part of traditional diets for centuries in Asia, but their concentrated extracts or use as nutraceuticals in Western countries raises new questions for food and drug regulators.

### 7.1. Regulatory Framework

Currently, no specific regulatory category exists for “postbiotics” as distinct entities in most jurisdictions [9,10]. Instead, algae-derived products are regulated either as foods, dietary supplements, or novel food ingredients. In the USA, for example, the FDA has not officially defined or addressed postbiotics yet, and would likely evaluate an algae-derived product under existing rules for food additives or supplements [9,94]. Several algae (e.g., kelp and nori) are Generally Recognized as Safe (GRAS) as foods. Alginate, carrageenan, and agar are approved as food additives. When it comes to health claims, structure-function claims (like “supports gut health” or “helps maintain healthy cholesterol levels”) can be made on supplements with proper disclaimers, but disease-risk reduction claims require significant scientific agreement or approval (e.g., in the EU via EFSA). The European Union, through EFSA, has a Novel Food regulation that some algae extracts might have to navigate (for instance, fucoxanthin-rich extracts could be considered novel, requiring safety dossiers).

Encouragingly, many marine-algal compounds edging into the market in Japan and Korea have been more proactive, with Japan allowing certain seaweed fibers in its FOSHU program. In Europe, some seaweed ingredients have novel food approval (e.g., there is an approved fucoidan extract for use in supplements). Postbiotics concept: It is worth noting that regulatory agencies are starting to discuss terms like probiotics, prebiotics, and postbiotics due to consumer interest. For example, an ISAPP consensus has been published to guide definitions [9], but regulators have yet to formally adopt these. Companies, at times, use “postbiotic” in marketing, but any functional claim has to be substantiated and not misleading. A potential advantage of postbiotics (like heat-killed microbes or fermented extracts) is that they avoid some of the regulatory hurdles of live microbes (which, in the EU, can be tricky since the term probiotic is considered a health claim itself). Marine microalgae, if pitched as postbiotics, might skip concerns about viability, focusing on their composition, which could simplify safety assessments. However, the lack of a dedicated framework means producers must ensure compliance through existing channels—essentially proving their product is safe as a food and effective for any claimed benefit through proper evidence.

### 7.2. Safety Issues

Generally, marine algae and their components are safe when consumed in traditional amounts, but there are notable considerations (Table 3):

**Heavy Metal Contamination:** Seaweeds can accumulate heavy metals from seawater, such as arsenic (particularly inorganic arsenic in some brown algae), cadmium, lead, and mercury [95]. In fact, dried hijiki (a brown seaweed) is known to contain inorganic arsenic above regulatory safety thresholds and acceptable daily intake limits, and some food agencies advise against its frequent consumption. Regulators have relatively few specific standards for heavy metals in seaweeds—in the US, for instance, no federal limits are set specifically for seaweed as a food commodity [96]. The European Union has begun to set some maximum levels: the EU has proposed limits for arsenic, cadmium, and lead in seaweed products. It is critical for manufacturers of algal supplements to source raw materials from clean waters and test for contaminants [97]. Many reputable companies do provide certificates of analysis for metal content. Novel processing methods (like washing or blanching seaweed) can reduce certain metals. Still, consumers should not greatly exceed recommended intakes. The risk is generally low, but chronic high consumption of contaminated seaweed could pose health risks (e.g., arsenic toxicity).

**Excess Iodine:** Marine algae, especially brown kelps (e.g., kombu), are extremely rich in iodine. Iodine is an essential nutrient for thyroid function, but *excessive intake can cause thyroid dysfunction*, either hypothyroidism or hyperthyroidism, depending on the individual’s baseline thyroid status [98]. There have been case reports of thyroid problems in people who overeaten kelp or taken high-dose kelp tablets. For example, a person developed hypothyroidism after consuming large amounts of kelp juice daily (due to iodine-induced thyroid inhibition) [98]. Regulatory bodies have taken note: France’s ANSES has warned consumers to be vigilant about excessive iodine from seaweed supplements, as it can cause cardiac or renal effects as well. Some countries recommend a limit on daily iodine from seaweed; for instance, the UK suggests not eating kelp daily or limiting portions. Manufacturers often put warning labels regarding iodine [99]. Balancing act: If algae are to be used for obesity, one must harness their fiber and bioactives without delivering toxic levels of iodine. This can be managed by choosing species with moderate iodine content (red and green algae typically have less iodine than brown) or by processing (boiling can leach out some iodine). Also, using extracts that exclude iodine (e.g., alginate extracts have negligible iodine) is another way. The lack of mandatory labeling of iodine content on seaweed products is a current gap—studies have highlighted that packaging often does not list iodine or heavy metals [100]. Regulators may need to update labeling requirements to include mineral content for consumer awareness.

**GI Tolerance:** As with any high-fiber substance, introducing large amounts of algal polysaccharides can cause gastrointestinal side effects in some individuals—bloating, gas, or mild diarrhea—due to fermentation. For example, an increase in bifidobacteria from seaweed fiber might transiently cause gas production. Generally, these symptoms are manageable and can be mitigated by gradual introduction. In human trials, 2–4 g/day of seaweed or 5–15 g/day of alginate were well tolerated [101]. In fact, alginate often relieves constipation by softening stools. Nonetheless, dose escalation and adequate hydration are advisable.

**Allergenicity:** Algae are not common allergens for ingestion (unlike shellfish or fish). However, there is potential for seafood-allergic individuals to react if impurities or due to cross-reactivity (some people allergic to seafood have reported issues with certain algae supplements). Also, some algae produce toxic metabolites in the wild (like certain microalgae can produce microcystins or BMAA toxin), so only cultivated, controlled strains (like commercial Spirulina, which is rigorously tested to be free of microcystins) should be used [102]. *Spirulina* has an excellent safety record, but contaminated batches (with toxic cyanobacteria) have caused issues historically, underscoring the need for quality control.

**Quality Control and Consistency:** Being natural products, the composition of algae can vary by harvest location, season, etc. Regulatory agencies will expect standardization if health claims are to be made. Companies might need to specify the content of active components (e.g., “X% fucoidan” or “Y mg fucoxanthin per dose”) to ensure batch-to-batch consistency. Good Manufacturing Practices (GMP) should be adhered to, especially for supplements, to avoid contaminants and ensure accurate labeling [103].

**Postbiotic Safety**: The concept of postbiotics implies that there are no live organisms, which generally is a safety plus (no risk of bacteremia in immunocompromised, etc.). Marine-algal postbiotics thus bypass concerns applicable to live probiotics. However, safety assessments still apply [104]. For example, a postbiotic preparation should be free of endotoxin contamination beyond what is expected in a food, and if heat-killed bacteria from fermentation are present, they should be from non-pathogenic, well-characterized strains. In the case of microalgae being considered postbiotic, one could argue their safety is supported by traditional use (Spirulina was consumed by the Aztecs, etc., and is GRAS) [94].

**Table 3:** Side Effects of Marine Algae Consumption.

Algae Type	Source	Side Effects	Dose Associated with Side Effects	References
Brown Algae (*Laminaria*, Kelp)	Kombu, Wakame, Hijiki	Excessive iodine intake leading to thyroid dysfunction (hyperthyroidism or hypothyroidism); iodine-induced thyrotoxicosis; potential heavy metal accumulation (arsenic, cadmium)	>1100 μg iodine/day (chronic intake)	[105,106,107]
Red Algae (*Porphyra*, Nori)	Nori sheets, dulse	Allergic reactions including urticaria, angioedema, and anaphylaxis; cross-reactivity with shellfish allergens; gastrointestinal discomfort	>15 g/day	[108,109]
Blue-Green Algae (*Spirulina*)	Dietary supplements, powder form	Contamination with microcystins causing hepatotoxicity; autoimmune reactions in susceptible individuals; nausea, diarrhea; potential phenylketonuria concerns due to phenylalanine content	Around 1 g daily for conditions like leukoplakia, up to 1500 mg daily for others.	[110,111]
Green Algae (*Chlorella*)	Chlorella supplements, tablets	Photosensitivity and skin reactions; gastrointestinal disturbances (bloating, gas, green discoloration of stool); potential exacerbation of immunodeficiency conditions	>16 g/day	[86]
Brown Algae (*Fucus vesiculosus*, Bladderwrack)	Weight loss supplements	Thyroid overstimulation; drug interactions with anticoagulants (high vitamin K content); acneiform eruptions; potential heavy metal toxicity	>600 μg iodine/day from supplements; vitamin K interactions with >500 mg/day	[100]
Red Algae (Carrageenan-producing)	Food additive, processed foods	Gastrointestinal inflammation and ulceration in animal studies; potential digestive issues; concerns about degraded carrageenan and colon cancer risk	Food-grade (undegraded) generally safe	[112]

Finally, from a regulatory outlook, as interest in postbiotics grows, we may see guidelines or monographs that include common algal postbiotics. The current lack of definition can cause some confusion in the marketplace (for instance, a product might call itself “postbiotic seaweed complex”—regulators would treat it essentially as a dietary fiber supplement). To gain consumer trust and regulatory acceptance, more clinical evidence demonstrating benefit is needed, which can then be used to substantiate claims legally. Safety-wise, the verdict is that marine-algal products are safe for the general population when sourced and used properly, but caution is needed regarding iodine and contaminants. Bodies like FAO/WHO have called for more data on seaweed food safety and the development of standards given the rising consumption [113].

In summary, marine-algal postbiotics hold a favorable safety profile and fall under existing food regulations, but improvements in regulatory clarity and product standardization are advisable. Ensuring that consumers get the metabolic benefits of these products without unintended side effects (like thyroid upset) will be key to their success as an accepted tool against obesity.

## 8. Conclusions and Future Directions

Marine algae offer a rich trove of bioactives that bridge nutrition and microbiome science, pointing to innovative strategies for obesity management. As reviewed, compounds derived from marine algae—“algal postbiotics”—can beneficially modulate the gut microbiota, leading to downstream improvements in the gut–adipose tissue axis. By nourishing beneficial bacteria and curbing detrimental ones, algae-derived fibers (fucoidan, alginate, carrageenan, ulvan, etc.) increase production of short-chain fatty acids and other metabolites that enhance gut barrier integrity, reduce systemic inflammation, and even promote adipose tissue browning. Simultaneously, algal polyphenols and carotenoids (phlorotannins, fucoxanthin, and astaxanthin, among others) directly target metabolic pathways in the host to reduce fat accumulation and oxidative stress in adipose depots. The net result, demonstrated in numerous animal studies and supported by early human trials, is an attenuation of obesity-related phenotypes: less weight gain, lower adiposity, improved insulin sensitivity, and a more anti-inflammatory status.

Crucially, this is achieved through natural and multifaceted mechanisms—essentially *using nutrition to tweak our microbiome allies*, who in turn help regulate our metabolism. Marine-algal postbiotics thus exemplify the power of diet-based interventions: they are lifestyle-friendly (can be incorporated into foods or taken as supplements), they address root causes of metabolic imbalance (like dysbiosis and inflammation), and they have a favorable safety profile when used judiciously. Moreover, they resonate with current consumer interest in plant-based and “ocean-friendly” solutions, possibly aiding adherence.

Of course, challenges and open questions remain. Human studies, while promising, are not yet abundant—more large-scale clinical trials are needed to confirm efficacy, optimal dosages, and long-term effects of various algae in diverse populations. The inter-individual variability in response should be investigated: factors such as baseline microbiota composition, genetics, and diet could influence how well someone responds to an algae intervention. For example, a person with low fiber intake may experience a bigger microbiota shift (and some GI discomfort at first) when adding seaweed, compared to someone already on a high-fiber diet. Personalized nutrition approaches could one day match specific algae (or their extracts) to individuals for maximal benefit.

From a translational perspective, ensuring consistent quality of algal products and monitoring for safety (especially iodine and heavy metals) will be paramount. Regulatory agencies are catching up with the trend, and we anticipate clearer guidelines on seaweed use in supplements and functional foods to emerge. The concept of postbiotics is gaining recognition, and marine-algal products could actually help drive this concept forward into regulatory acceptance, given their non-live nature and health benefits.

In conclusion, the intersection of marine biology and gut microbiology opens a new frontier in tackling obesity—one where the ocean’s harvest helps to recalibrate our internal microbial universe for better health. Marine-algal-derived postbiotics represent a compelling, natural, and sustainable approach to modulate the gut–adipose tissue axis. They add to our arsenal a preventive/therapeutic option that is not a drug with a single target, but a holistic agent working through the microbiome to restore metabolic balance. As we continue to grapple with the global obesity epidemic, such innovative solutions rooted in nature and ecology are timely and welcome. Harnessing the power of marine algae could improve both gut health and weight management outcomes across diverse populations in the years to come, offering a rising tide of metabolic benefits.

## Figures and Tables

**Table 1:** Gut microbiota–adipose tissue axis in obesity.

Component	Role in Obesity	Mechanism	References
*Firmicutes*/*Bacteroidetes* Ratio	Increased ratio associated with obesity and enhanced energy harvest	Increased capacity for polysaccharide fermentation and SCFA production; enhanced caloric extraction from diet	[18,19]
Short-Chain Fatty Acids (SCFAs)	Dual role: energy source and metabolic regulators	Acetate, propionate, and butyrate serve as substrates for lipogenesis; activate GPR41/GPR43 receptors; influence satiety hormones (GLP-1, PYY)	[20,21]
Lipopolysaccharide (LPS)	Promotes metabolic endotoxemia and low-grade inflammation	Increased gut permeability leads to LPS translocation; activates TLR4 signaling in adipocytes; induces inflammatory cytokine production	[22,23,24]
*Akkermansia muciniphila*	Protective against obesity and metabolic dysfunction	Strengthens gut barrier integrity; reduces inflammation; improves glucose homeostasis; increases mucus layer thickness	[4,25,26]
Bile Acids	Regulate lipid and glucose metabolism	Modified by gut bacteria; act as signaling molecules via FXR and TGR5 receptors; influence energy expenditure in adipose tissue	[27,28]
Gut Barrier Dysfunction	Increased permeability promotes obesity-related inflammation	Disrupted tight junctions allow bacterial translocation; triggers immune activation in adipose tissue; promotes insulin resistance	[29]
Microbial Metabolites (Branched-Chain Amino Acids)	Elevated levels associated with insulin resistance and obesity	Produced by specific bacterial taxa; interfere with insulin signaling; promote inflammation in adipose tissue	[30]
*Lactobacillus* and *Bifidobacterium* species	Generally protective; reduced abundance in obesity	Produce antimicrobial compounds; strengthen gut barrier; reduce inflammation; modulate fat storage	[31]
Trimethylamine-N-oxide (TMAO)	Pro-obesogenic and pro-inflammatory metabolite	Derived from dietary choline/L-carnitine via microbial TMA production; promotes adipose tissue inflammation and insulin resistance	[32]
Adipose Tissue Macrophages	Infiltration increased by microbiota-derived signals	LPS and other bacterial products activate inflammatory pathways; M1 polarization promotes insulin resistance; chronic inflammation perpetuates obesity	[33]
Gut-Brain-Adipose Axis	Microbiota influences appetite and fat distribution via neural pathways	Bacterial metabolites affect vagal signaling; modulate hypothalamic appetite centers; influence sympathetic nervous system activity in adipose tissue	[34]
Mucin-Degrading Bacteria	Increased abundance in obesity disrupts gut barrier	Erosion of mucus layer increases host–microbiota proximity; facilitates LPS translocation and inflammation	[35]

Abbreviations: FXR (Farnesoid X Receptor); GLP-1 (Glucagon-Like Peptide-1); GPR41 (G-Protein-Coupled Receptor 41); PYY (Peptide YY); SCFA (short-chain fatty acid); TGR5 (Takeda G-protein 5); TLR4 (toll-like receptor 4); TMA (Trimethylamine).

**Table 2:** Summary of key human clinical trials on marine algae and obesity-related outcomes.

Algae Type	Dose	Duration	Sample Size	Key Findings	References
*Himanthalia elongata* (whole brown seaweed)	2 g/day	30 days	40 overweight adults	↑ *Parabacteroides*, *Bacteroides*, SCFA-producers; improved microbiota diversity	[7]
Alginate	15 g/day	14 weeks	96 overweight/obese adults	Significant weight loss (−1.8 kg vs. placebo); altered gut microbiota; ↑ fecal bile acid excretion	[90]
*Spirulina*	1–8 g/day	8–24 weeks	11 RCTs, 450 participants	Mean weight reduction: −1.56 kg; greater effect in obese (−2.0 kg); ↓ body fat %, ↓ waist circumference	[37]
Fucoxanthin	12 mg/day	12 weeks	28 metabolic syndrome patients	Weight loss: −1.5 kg; ↓ BMI, ↓ waist; improved insulin sensitivity; ↓ blood pressure and triglycerides	[61]
*Ascophyllum nodosum* enriched bread	4% seaweed	6 weeks	38 overweight males	↓ subsequent energy intake; ↑ *Bifidobacterium*; improved satiety	[92]
Rhamnan sulfate (green algae)	100 mg/day	2 weeks	24 adults with constipation	↑ stool frequency; ↑ *Bacteroidetes*, ↓ *Clostridia*; improved GI function	[72]

Abbreviations: ↑ = increase; ↓ = decrease; SCFA = short-chain fatty acid; RCT = randomized controlled trial; GI = gastrointestinal.

## Data Availability

No data were produced from this study; all data used are contained in this article and published papers in the references.

## Abbreviations

SCFA	Short-Chain Fatty Acid
LPS	Lipopolysaccharide
TLR4	Toll-Like Receptor 4
FXR	Farnesoid X Receptor
TGR5	Takeda G-Protein Receptor 5
GPR43/FFAR2	G-Protein Coupled Receptor 43/Free Fatty Acid Receptor 2
GPR41/FFAR3	G-Protein Coupled Receptor 41/Free Fatty Acid Receptor 3
GLP-1	Glucagon-Like Peptide-1
PYY	Peptide YY
HFD	High-Fat Diet
BMI	Body Mass Index
UCP1	Uncoupling Protein 1
PPARγ	Peroxisome Proliferator-Activated Receptor Gamma
C/EBPα	CCAAT/Enhancer-Binding Protein Alpha
SREBP-1c	Sterol Regulatory Element-Binding Protein 1c
TMAO	Trimethylamine-N-oxide
RS	Rhamnan Sulfate
UAOS	Unsaturated Alginate Oligosaccharides
F/B Ratio	Firmicutes/Bacteroidetes Ratio
GRAS	Generally Recognized as Safe
FOSHU	Food for Specified Health Use
RCT	Randomized Controlled Trial
PUFA	Polyunsaturated Fatty Acid
FAO	Food and Agriculture Organization
WHO	World Health Organization
ISAPP	International Scientific Association for Probiotics and Prebiotics
GI	Gastrointestinal
